# A New Self-Powered Sensor Using the Radial Field Piezoelectric Diaphragm in d_33_ Mode for Detecting Underwater Disturbances

**DOI:** 10.3390/s19040962

**Published:** 2019-02-24

**Authors:** Xingxu Zhang, Xiaobiao Shan, Zhiyuan Shen, Tao Xie, Jianmin Miao

**Affiliations:** 1 State Key Laboratory of Robotics and System, Harbin Institute of Technology, Harbin 150001, China; xingxuz@163.com (X.Z.); shanxiaobiao@hit.edu.cn (X.S.); 2 School of Mechanical and Aerospace Engineering, Nanyang Technological University, Singapore 639798, Singapore; 3 School of Biological Sciences, University of Bristol, Bristol BS8 1QU, UK; zhiyuan.shen@bristol.ac.uk

**Keywords:** radial field diaphragm, piezoelectric sensors, underwater sensing, micro-fabrication, finite element analysis

## Abstract

This paper presents a new sensor based on a radial field bulk piezoelectric diaphragm to provide energy-efficient and high-performance situational sensing for autonomous underwater vehicles (AUVs). This sensor is self-powered, does not need an external power supply, and works efficiently in d_33_ mode by using inter-circulating electrodes to release the radial in-plane poling. Finite element analysis was conducted to estimate the sensor behavior. Sensor prototypes were fabricated by microfabrication technology. The dynamic behaviors of the piezoelectric diaphragm were examined by the impedance spectrum. By imitating the underwater disturbance and generating the oscillatory flow velocities with a vibrating sphere, the performance of the sensor in detecting the oscillatory flow was tested. Experimental results show that the sensitivity of the sensor is up to 1.16 mV/(mm/s), and the detectable oscillatory flow velocity is as low as 4 mm/s. Further, this sensor can work well under a disturbance with low frequency. The present work provides a good application prospect for the underwater sensing of AUVs.

## 1. Introduction

Nowadays, autonomous underwater vehicles (AUVs) which can travel underwater without requiring input from an operator play an important role in underwater exploration and military applications [1]. Traditionally sensing strategies of AUVs to perceive their surroundings are sound navigation and ranging (SONAR) and optical imaging [2]. However, sonar technology has a blind zone and the intense sound waves can be fatal to aquatic animals [3,4]. Optical imaging will fail to work in dark and turbid water [5]. Besides, they usually have a large size and heavy weight [6,7].

Recently, researchers have been paying attention to eliminating the issues of AUVs mentioned above by taking advantage of sensing principles inspired by Nature. The lateral line of blind fish is the most studied one. Though it is blind, the blind fish is still capable of perceiving its surroundings and avoiding obstacles by relying on its lateral line [8]. The inspiration has led to the design of novel sensors. The sensing elements of the devices typically consist of deformable flexural elements, such as cantilever beams, diaphragms and bridges [9]. Peleshanko, et al. [10] utilized SU-8 standing structures on thin silicon cantilever beams. Kottapalli, et al. [11,12] fabricated polymer MEMS sensors with a membrane structure to detect the moving of underwater objects. Kottapalli, et al. [13,14] also designed a few sensors with a standing pillar which can extend into the ambient flow. However, these flow sensors are piezoresistive devices which need a power supply to bias them.

Piezoelectric materials are a good solution to this problem. They have been widely used in applications such as energy harvesters [15,16,17] and accelerometers [18]. Charges will be induced on the surface when it is exposed to a deformation so one can collect these charges to get information about the excitation. When a piezoelectric material is utilized to sense, it is self-powered. Asadnia et al. [19,20] have conducted some studies on micromachined piezoelectric sensors for flow sensing. The PZT layer was fabricated by a sol-gel method and patterned electrodes were completed by microfabrication techniques. The PZT elements of the sensors work in d_31_ mode which is not efficient compared with d_33_ mode because usually the d_33_ parameter of PZT is two times larger than the d_31_ parameter. For micromachined piezoelectric circular diaphragms, it is usually hard to do in-plane poling because the radial scale is much bigger than the thickness. Bryant et al. [21,22] realized an equivalent d_33_ mode radial field diaphragm which can solve the problem. It can be poled by inter-circulating electrodes or interdigitated ring electrodes. Based on this research, Hong et al. [23,24] released a thin film PZT diaphragm by ring shaped interdigitated electrodes. Wang et al. [25] reported a bulk PZT diaphragm with a ring shaped interdigitated electrode. Besides, there are also some studies which design a piezoelectric diaphragm bearing double side spiral electrodes [26,27]. However, most previous research with respect to the radial field piezoelectric diaphragm has focused on actuating or high frequency sensing.

Therefore, this paper presents a new sensor based on the radial field piezoelectric diaphragm for underwater sensing applications in AUVs with low frequency signals. Finite element analysis was performed to estimate the behavior of the sensor. Inter-circulating electrodes were fabricated by microfabrication techniques. The impedance spectrum of the diaphragm was examined which demonstrated a satisfactory dynamic behavior. The sensor’s ability to detect the low frequency oscillatory flow velocity was illustrated.

## 2. Design and Simulation

**Figure 1:**
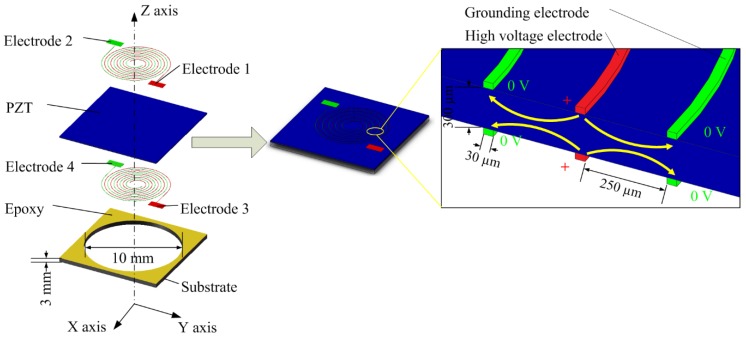
Structural diagram of a radial field piezoelectric diaphragm and its polarization distribution.

Figure 1 shows the structural diagram of a radial field piezoelectric diaphragm and its polarization distribution. Inter-circulating electrodes were adopted here to supply a DC voltage for the piezoelectric material poling and collect the charges induced by the piezoelectric material when the diaphragm worked as a sensor.

With the inter-circulating electrodes on both sides of the piezoelectric ceramic, when a DC voltage is applied to the electrodes to do the in-plane poling, the result is a radially distributed electric field. The electrode wire width is 30 μm and the space between adjacent electrodes is 250 μm. The radius of the smallest semicircle is 1550 μm and there are 11 turns of the semicircular rings. By bonding the bulk piezoelectric ceramic to a through-hole substrate using epoxy, a radial field piezoelectric diaphragm will be obtained which can work in d_33_ mode. In contrast to the thin film deposition piezoelectric diaphragm which has to be deposited on some substrate and form multiple layers, our design can avoid this complex structure. Besides, both sides of the electrodes are uncovered so that the PZT etching to contact the bottom electrodes is avoided. Moreover, by using the radial field piezoelectric diaphragm, the designed sensor works based on the piezoelectric effect sensing principle. When the movement of underwater objects generates disturbances that cause a pressure on the piezoelectric diaphragm, the piezoelectric diaphragm will bend and generate electric charges. By collecting the generated electric charges using electrodes, the information of the underwater disturbances will be obtained. The sensor does not need an external power supply and hence it is self-powered.

**Figure 2:**
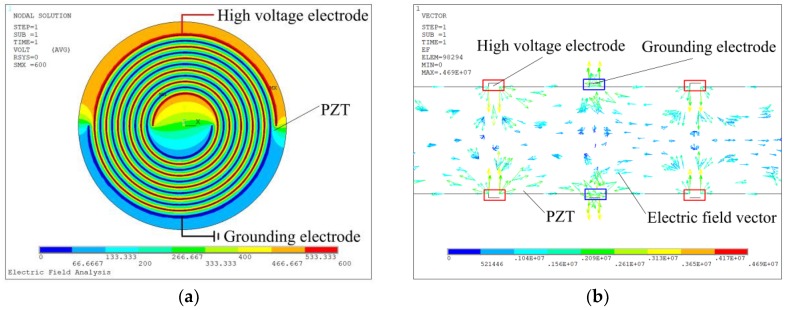
Electrostatic field simulation results: (**a**) Contour of poling electric potential distribution on the surface of the diaphragm. (**b**) Poling electric field vectors distribution on one cross section of the diaphragm.

In order to illustrate the effect of the inter-circulating electrodes on the distribution of the electric field and analyze the properties of the sensor, a model with the geometrical parameters mentioned above was built to conduct the finite element analysis. A circular piezoelectric diaphragm with clamped boundary condition can be adopted so that the epoxy layer and the substrate are not needed to be built in the finite element analysis. The circular piezoelectric diaphragm is 300 μm thick and its diameter is 10 mm. The electrostatic field analysis was employed to calculate the electric field vectors during the poling process. The element SOLID 122, which is a 3-D, 20-node, and charge-based electric element, was used to conduct the electrostatic field analysis. There are four independent electrodes on the diaphragm, named as Electrode 1, Electrode 2, Electrode 3 and Electrode 4 (as shown in Figure 1). When the poling process is conducted, Electrode 1 and Electrode 3 are wire-connected as one contact port and Electrode 2 and Electrode 4 are wire-connected as another. A 600 V biased voltage was applied between these two ports to generate the poling electric field. Figure 2 shows the finite element simulation results. Figure 2a shows the contour of the poling electric potential distribution on the surface of the piezoelectric diaphragm and Figure 2b shows the poling electric field vectors distribution on one cross section of the diaphragm. From Figure 2, one can know that the poling electric field between two electrodes lies uniformly in the in-plane direction.

Then the simulation results of the electric field strength distribution in each element were extracted. The electric field strength in each element will be compared with the coercive field strength (5 kV/cm for the PZT used). If the electric field strength was smaller than the coercive field strength, the element coordinate system maintained its direction; otherwise the element coordinate system was converted to make the z-axis direction parallel with the poling field vector. The reorientation result was written as a macro command which can be invoked during the piezoelectric modal and static analysis.

The element SOLID 226, which is a 3-D, 20-node, and coupled-field element, was used to conduct the piezoelectric modal analysis and static analysis. The piezoelectric parameters set in the simulation were the same as the parameters of the material which was lead zirconate titanate (PZT 5A, Fuji Ceramics Co., Fujinomiya, Japan), as follows:

Elastic compliance matrix:

$$\left[\begin{array}{cccccc} s_{11}^{E} & s_{12}^{E} & s_{13}^{E} & 0 & 0 & 0\\ s_{12}^{E} & s_{11}^{E} & s_{13}^{E} & 0 & 0 & 0\\ s_{13}^{E} & s_{13}^{E} & s_{33}^{E} & 0 & 0 & 0\\ 0 & 0 & 0 & s_{44}^{E} & 0 & 0\\ 0 & 0 & 0 & 0 & s_{55}^{E} & 0\\ 0 & 0 & 0 & 0 & 0 & s_{66}^{E} \end{array}\right]=\left[\begin{array}{cccccc} 15.4 & -4.8 & -8.4 & 0 & 0 & 0\\ -4.8 & 15.4 & -8.4 & 0 & 0 & 0\\ -8.4 & -8.4 & 15.4 & 0 & 0 & 0\\ 0 & 0 & 0 & 47.8 & 0 & 0\\ 0 & 0 & 0 & 0 & 47.8 & 0\\ 0 & 0 & 0 & 0 & 0 & 40.4 \end{array}\right]\times 10^{-12}\;m^{2}N^{-1}$$

Piezoelectric constant matrix:

$$\left[\begin{array}{cccccc} 0 & 0 & 0 & 0 & d_{15} & 0\\ 0 & 0 & 0 & d_{15} & 0 & 0\\ d_{31} & d_{31} & d_{33} & 0 & 0 & 0 \end{array}\right]=\left[\begin{array}{cccccc} 0 & 0 & 0 & 0 & 590 & 0\\ 0 & 0 & 0 & 590 & 0 & 0\\ -191 & -191 & 430 & 0 & 0 & 0 \end{array}\right]\times 10^{-12}\;{\mathrm{CN}}^{-1}$$

Permittivity matrix:

$$\left[\begin{array}{ccc} \varepsilon_{11}^{T} & 0 & 0\\ 0 & \varepsilon_{11}^{T} & 0\\ 0 & 0 & \varepsilon_{33}^{T} \end{array}\right]=\left[\begin{array}{ccc} 1780 & 0 & 0\\ 0 & 1780 & 0\\ 0 & 0 & 1950 \end{array}\right]$$

Firstly, the polarization direction in each element was set as the identical direction with the z-axis direction of the local element coordinate system, which reflected the thickness poling condition of the purchased piezoelectric wafer. Then the macro command mentioned before in the electrostatic field simulation was invoked and hence the reorientation of the polarization was achieved. A modal analysis was conducted after the polarization reorientation and the edge was set as clamped boundary condition. 

**Figure 3:**
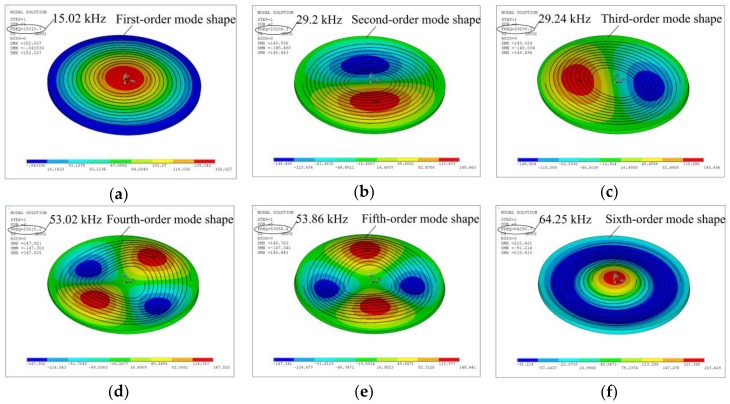
Modal shapes simulated by modal analysis: (**a**) f_1_ = 15.02 kHz, (**b**) f_2_ = 29.20 kHz, (**c**) f_3_ = 29.24 kHz, (**d**) f_4_ = 53.02 kHz, (**e**) f_5_ = 53.86 kHz, (**f**) f_6_ = 64.25 kHz.

Figure 3 shows the first six resonances of the radial field piezoelectric diaphragm. According to the modal analysis, one can know about the dynamic behavior of the sensor and its fundamental resonant frequency is around 15.02 kHz.

**Figure 4:**
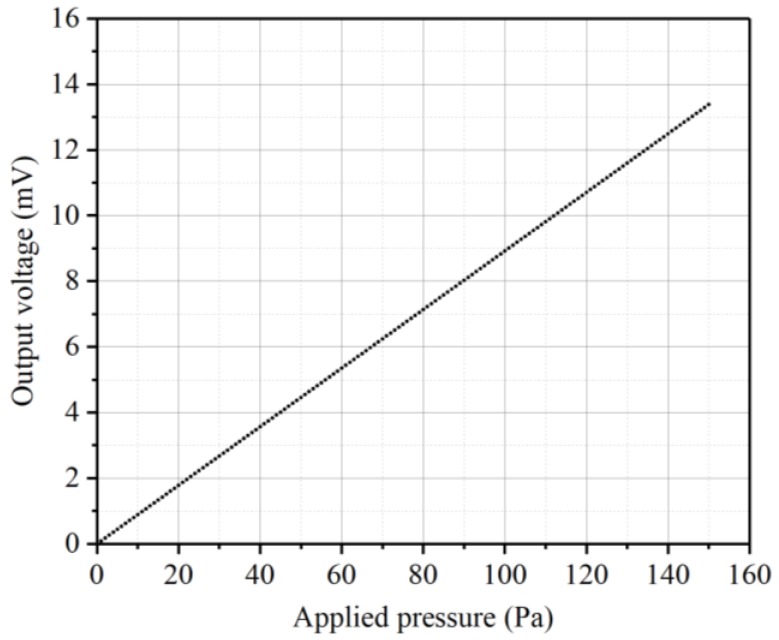
Piezoelectric static analysis simulation results.

Since disturbances generated by underwater moving objects are usually not higher than 100 Hz, the designed sensor will work at low frequencies under most of the circumstances. Therefore, the piezoelectric diaphragm will have a quasi-static response. Piezoelectric static analysis was performed to estimate the sensor voltage output when applied pressures to the diaphragm. Figure 4 shows the piezoelectric static analysis simulation results. When a static pressure of 1 Pa is applied to the diaphragm, the sensor voltage output is 0.089 mV.

## 3. Fabrication Using the Microfabrication Technology

**Figure 5:**
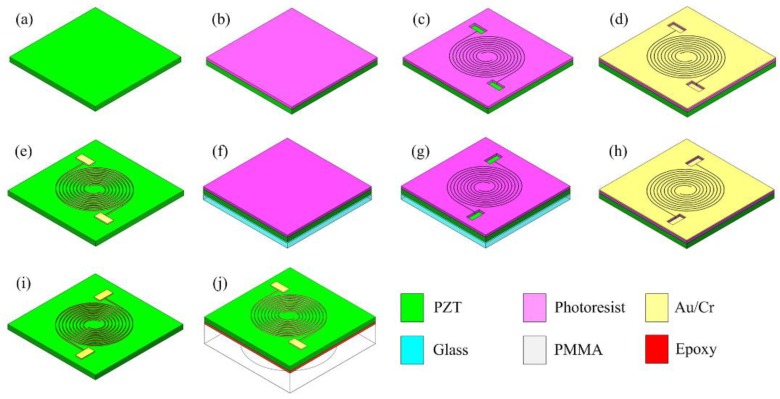
Schematics of the fabrication procedure. (**a**) PZT wafer; (**b**) Spin coating photoresist; (**c**) Lithography and photoresist patterning; (**d**) Magnetron sputtering gold with chromium as electrodes; (**e**) Lift-off process; (**f**) Bonding with glass and backside spin coating photoresist; (**g**) Alignment lithography; (**h**) Magnetron sputtering gold with chromium as backside electrodes; (**i**) Lift-off process; (**j**) Bonding with a through hole PMMA to release a diaphragm.

Figure 5 is the schematics of the fabrication procedure. Inter-circulating electrodes on the surface of the PZT were fabricated by MEMS microfabrication technology in a clean room.

The PZT wafer (circular disk wafer, PZT 5A, Fuji Ceramics Co, thickness = 300 μm) was first cleaned by soaking and rinsing it in acetone and isopropyl alcohol (IPA) for several minutes. The top side of the PZT was spin coated with 7 μm thick AZ9260 photoresist (Figure 5b) and then it was prebaked at 110 degrees Celsius for 4 min. The photoresist was exposed in a MA6 mask aligner (Karl Suss, Garching, Germany) for 50 s and developed in an AZ400K photoresist developer (Merck Performance Materials, Darmstadt, Germany) for 60 s (Figure 5c). The PZT with patterned photoresist was sputtered 400 nm thick gold with 40 nm thick chromium as the buffer layer (Figure 5d). Then the wafer was soaked in acetone for 10 h to carry out lift-off process. The metal layer which was adhered on the surface of photoresist would automatically peel off, however the metal layer deposited on the PZT can remain on its surface (Figure 5e). After the top side lift-off process, the PZT wafer was bonded with a transparent glass wafer by 5.2 μm photoresist and the back side of the PZT was spin coated with 7 μm thick AZ9260 photoresist again to prepare for the backside lithography (Figure 5f). The glass wafer can assist the backside lithography alignment. Lithography (Figure 5g), magnetron sputtering (Figure 5h) and the lift-off process (Figure 5i) were conducted again on the backside of the PZT wafer. During the lift-off process, the acetone will dissolve the photoresist and the glass will be removed from the PZT wafer. Finally, we got a PZT with patterned electrodes on both sides but without any other layers on it. The PZT wafer will be diced by automatic dicing machine to separate each device. Each device was bonded with a 3 mm thick polymethyl methacrylate (PMMA) plate which had a laser-drilled hole. A non-conductive epoxy (EPO-TEK-H70E, Epoxy Technology Inc, Billerica, MA, USA) was used to bond the PZT with PMMA. The device was then heat-treated for 12 h at 55 degrees Celsius to allow the epoxy to cure and make a strong bond. After the bonding was finished, the piezoelectric diaphragm was released.

**Figure 6:**
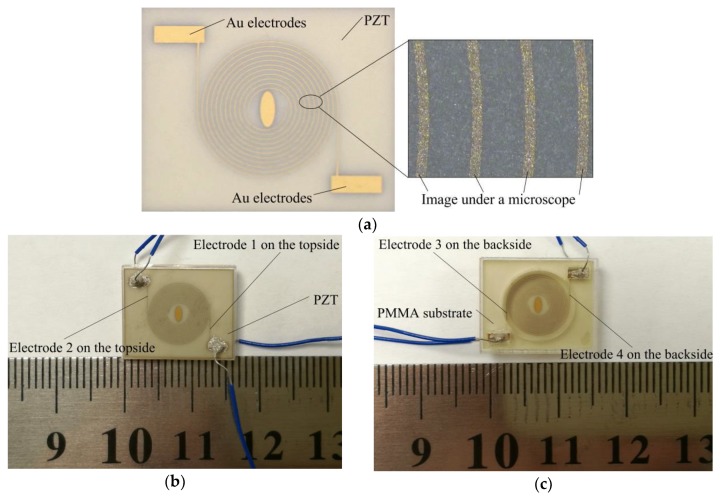
Fabricated sensor: (**a**) Electrode pattern on the PZT; (**b**) Top side of the fabricated sensor; (**c**) Back side of the fabricated sensor.

Figure 6a shows a photograph of the electrode pattern on the PZT. The ellipse in the center is used to distinguish the diaphragm center and the direction of the electrodes. Figure 6b shows the top side of the fabricated sensor and Figure 6c shows its back side.

The electrode contact pads were connected to wires by using conductive epoxy (EPO TEK EJ2189-LV). The device was then heat-treated for 6 h at 55 degrees Celsius to cure and make the connections dry. A model 610E high voltage supply (TREK, New York, NY, USA) was used to provide the high DC voltage for material poling. In order to avoid short circuit, devices were soaked in insulating oil during the poling process. Devices were poled at 600 V for 30 min at the room temperature. Hence, the radial field piezoelectric diaphragm was released.

## 4. Results and Discussion

### 4.1. Impedance Spectrum of the Radial Field Piezoelectric Diaphragm

**Figure 7:**
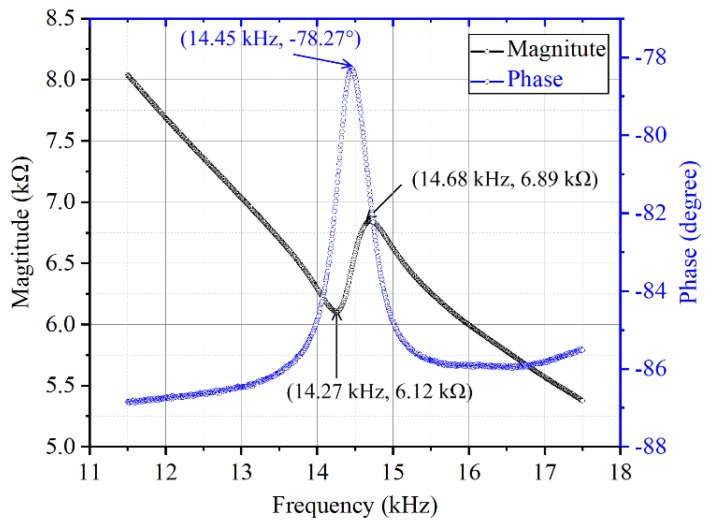
Impedance spectrum of the radial field diaphragm.

**Figure 8:**
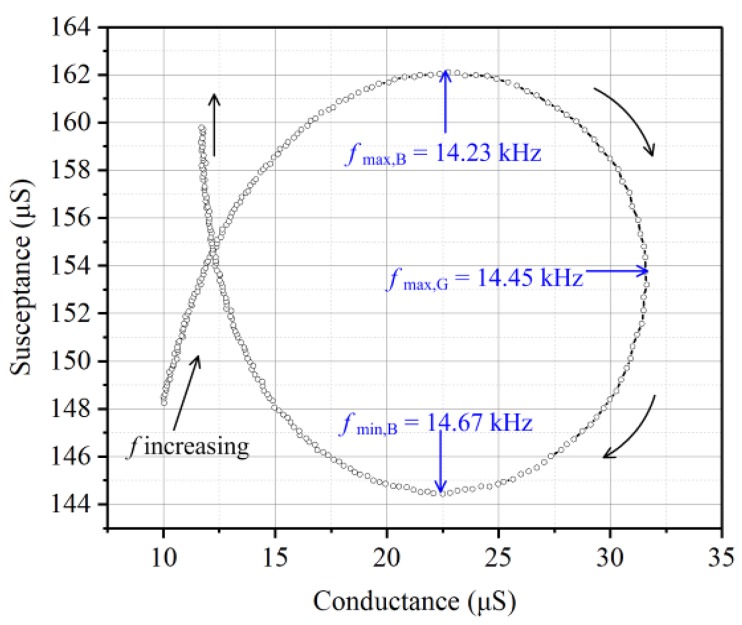
Admittance locus of the radial field diaphragm.

The resonant frequency behavior of the diaphragm was characterized by using an impedance analyzer (Agilent 4294A, Agilent Inc., Bayan, Malaysia). Figure 7 shows the impedance spectrum of the diaphragm around the first resonance in the air. The fundamental resonance frequency is 14.45 kHz and the result agrees with the simulation modal analysis results. Figure 8 shows the admittance locus of the diaphragm around the first resonance in the air. The results were tested in the condition that Electrode 1 and Electrode 4 were connected but Electrode 2 and Electrode 3 were connected.

The electromechanical performance of a piezoelectric transducer usually can be characterized by the coupling coefficient *k*^2^ which is a measure of the convertible energy between mechanical and electrical domains. It can be calculated by [26]:

(1) $$k^{2}=1-{\left(\frac{f_{s}}{f_{p}}\right)}^{2}$$

where *f_p_* is the parallel resonance frequency, *f_s_* is the series resonance frequency. From Figure 7, one can get the values of *f_p_* and *f_s_*. The calculated coupling coefficient is 5.51%. 

The mechanical loss of the resonator can be measured by the dimensionless quality factor *Q*. Bigger quality factor means lower mechanical loss. It can be derived by [26]:

(2) $$Q=\frac{f_{\max,G}}{f_{\min,B}-f_{\max,B}}$$

where *f*_max,*G*_ is the frequency at the maximum conductance, *f*_max,*B*_ is the frequency at the maximum susceptance and *f*_min,*B*_ is the frequency at the minimum susceptance. From Figure 8, one can get the values of *f*_max,*G*_, *f*_max,*B*_ and *f*_min,*B*_. The calculated quality factor is 32.84. The coupling coefficient and quality factor demonstrate a good dynamic behavior of the radial field diaphragm. The sensitivity of the sensor in this paper, which is 0.089 mV/Pa, is 13.69 times that of the d31 mode diaphragm reported by Wang, et al. [28].

Some detailed corresponding comparisons are listed in Table 1.

In order to examine the dynamic behavior of the sensor in water, the phase spectrum of the diaphragm in water was also tested by an impedance analyzer (Agilent 4294A, Agilent Technologies Inc., Santa Clara, CA, USA). The depth when measuring the dynamic behavior of the sensor in water is 60 mm. The Electrode 1 and Electrode 2 were connected to the impedance analyzer. 

**Figure 9:**
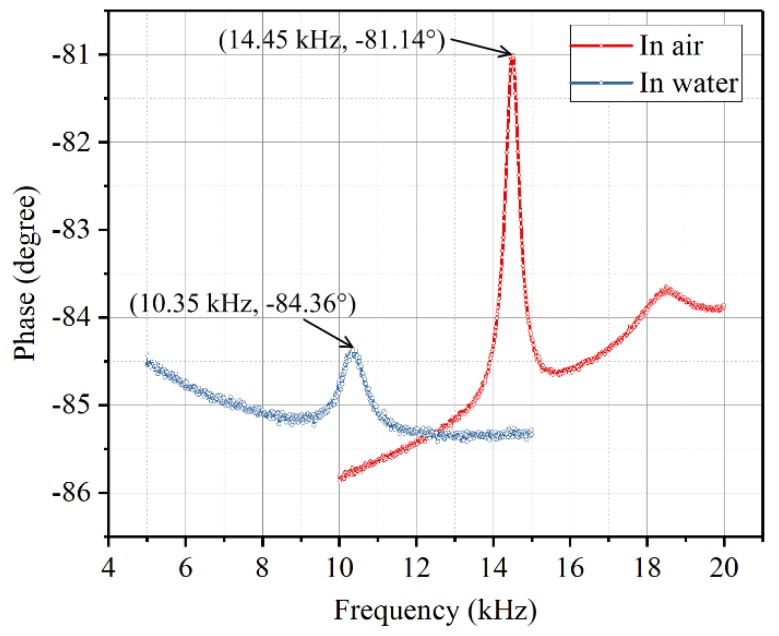
Phase spectrum of the diaphragm in air and water.

Figure 9 shows the phase spectrum changes when the sensor was put in water in contrast to in air. One can know that the resonant frequency and the phase peak of the diaphragm decreased when the diaphragm was put in water. This is because the vibrating diaphragm sets the surrounding fluid into motion and the fluid generates an added mass and damping forces on the diaphragm. Even so, the diaphragm still has a good dynamic behavior and can work well in water.

### 4.2. Oscillatory Flow Velocity Sensing

In most of the presented experiments, a vibrating sphere was used to apply a hydrodynamic excitation and imitate an underwater disturbance [19]. Hence, we used a vibrating sphere in water to generate disturbance and test the sensor response. Since disturbances generated by underwater moving objects are usually lower than 100 Hz, most experiments in this paper were conducted at low frequencies.

A stainless steel sphere of 16 mm diameter was connected to a permanent magnet mini-shaker (model 4810, B&K, Norcross, GA, USA) by a 2 mm diameter stainless steel rod. The mini-shaker was driven by sinusoidal signals so that the sphere can vibrate and generate the underwater stimulus. When the input signals have different amplitudes and frequencies, the mini-shaker can consequently vibrate at different velocities. Hence, the vibrating sphere can generate different oscillating flow velocities when it is put in water. 

**Figure 10:**
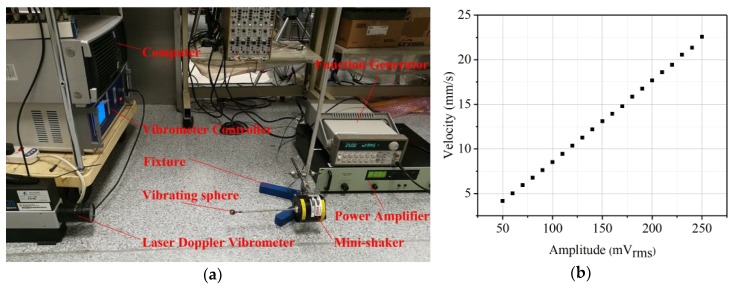
Mini-shaker calibration. (**a**) Photograph of the LDV testing; (**b**) Vibration velocity of the vibrating sphere as a function of driving amplitude at a constant frequency of 35 Hz.

Before testing the sensor response to the underwater disturbance generated by the vibrating sphere, we need to know its vibration velocities for various driving amplitudes. A LV-S01 model laser doppler vibrometer (LDV, Sunny Instruments, Singapore) was used to calibrate the vibration velocities of the vibrating sphere. Here the frequency of 35 Hz was selected to do the testing. A function generator (Agilent 33120A, Agilent Technologies Inc,) was used to supply signals. The signals were amplified by a specified gain of 20 dB through a power amplifier (type 2718, B&K) and then were used to drive the mini-shaker. Figure 10a shows a photograph of the calibrating experiment. Figure 10b shows the calibration results. The transverse axis represents the voltage amplitude from the signal generator and the vertical axis represents the vibration velocities of the vibrating sphere. The vibration velocity of the vibrating sphere increases linearly with increasing amplitude when the mini-shaker is set at a constant frequency.

**Figure 11:**
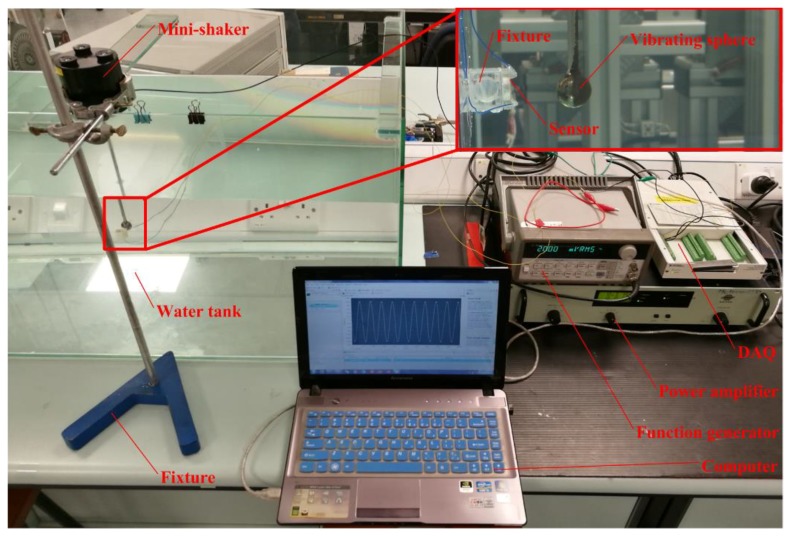
Underwater disturbance sensing experiment system.

**Figure 12:**
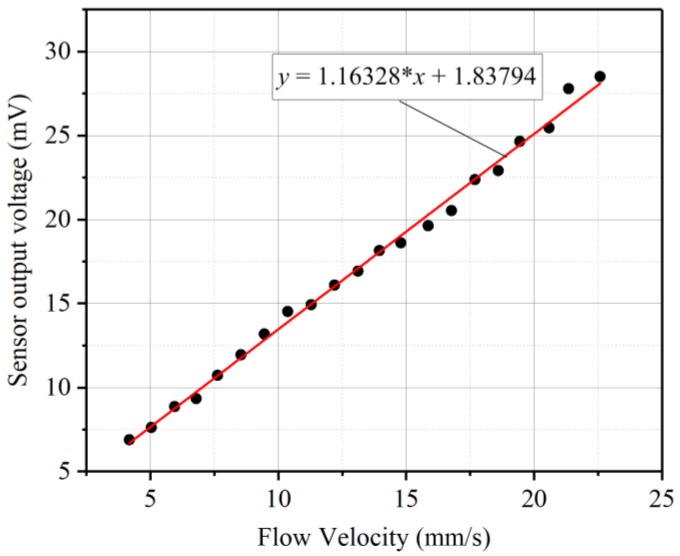
Experimental results of underwater oscillatory flow velocity sensing.

After the velocity of the vibrating sphere was calibrated, the underwater testing of the sensor was carried out. The sensor surfaces were coated a thin film of silicone so that it can be insulated from water. Figure 11 shows a photograph of the underwater disturbance sensing experiment system. The sensor was mounted on the wall of a water tank of dimensions 1 × 0.45 × 0.45 m^3^ and the mini-shaker was mounted on the top of the water tank. The input signal which was used to drive the mini-shaker was provided by the signal generator and amplified by the power amplifier. The sphere connected to the mini-shaker was immersed into water and positioned right in front of the sensor diaphragm at the distance of 30 mm. The vibrating sphere generated oscillatory flow velocities in water. The oscillatory flow generated by the stimulus caused a deflection of the diaphragm and the sensor can generate an output voltage. The output from the sensor was acquired by a National Instruments data acquisition card (NI-DAQ USB-6289, National Instruments, Austin, TX, USA) at a rate of 2 kHz and recorded in the software LabVIEW. Figure 12 shows the experimental results of the sensor response to the oscillatory flow velocities in water. The oscillatory flow velocities can refer to the LDV results before. The sensor output is the peak to peak value of the sinusoidal voltage response. The sensor shows a linearly increasing output with respect to increasing oscillatory flow velocity amplitudes. It demonstrates a high sensitivity of 1.16 mV/(mm/s) and can detect oscillatory flow velocity as low as 4 mm/s.

### 4.3. Low Frequency Sensing

In order to demonstrate sensor’s ability of response to low frequency disturbance, low frequency testing was conducted. The experiment set up was similar with the oscillatory flow velocity sensing experiments’ except that the mini-shaker was driven at the fixed amplitude of 250 mV_rms_ but at frequencies from 10 to 200 Hz. 

**Figure 13:**
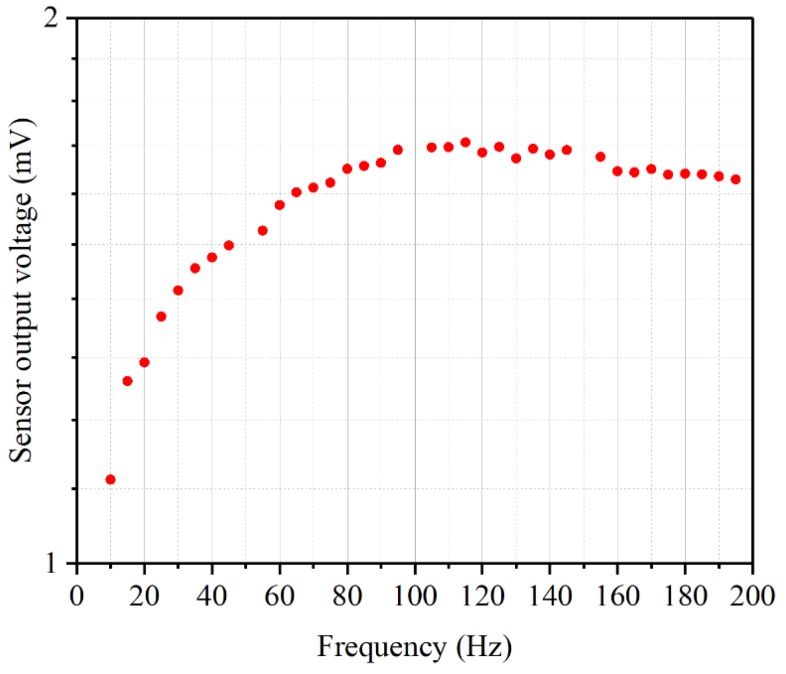
Frequency response in water for low frequency signals by using a vibrating sphere.

Figure 13 shows the peak to peak voltage amplitude output of the sensor obtained by the data acquisition card as the vibrating sphere with frequencies from 10 to 200 Hz. It can be observed that the sensor output increases first and then decreases with respect to the increasing oscillatory flow velocity frequencies. This is due to the specific performance of the mini-shaker. When a load is applied to the mini-shaker, when the frequency is lower than a certain frequency, the output acceleration amplitude of the mini-shaker increases with the increasing of frequency. Hence the output velocity amplitude of the mini-shaker also increases. However, when the frequency is higher than a certain frequency, the output acceleration amplitude of the mini-shaker is roughly stable so that the output velocity amplitude will decrease with the increasing of frequency. The sensor output agrees with the stimulus changes decided by the mini-shaker properties. And the low frequency testing results demonstrate that the designed sensor can work well under low frequency disturbance.

### 4.4. Object Location Sensing

This section through experiments illustrated the ability of the designed sensor in localizing a vibrating sphere in water. The vibrating sphere was driven at the fixed frequency of 35 Hz and fixed amplitude of 250 mV_rms_, while its position was shifted vertically and in direction of the sensor. The signals from the sensor were acquired using the data acquisition card and recorded in LabVIEW. 

**Figure 14:**
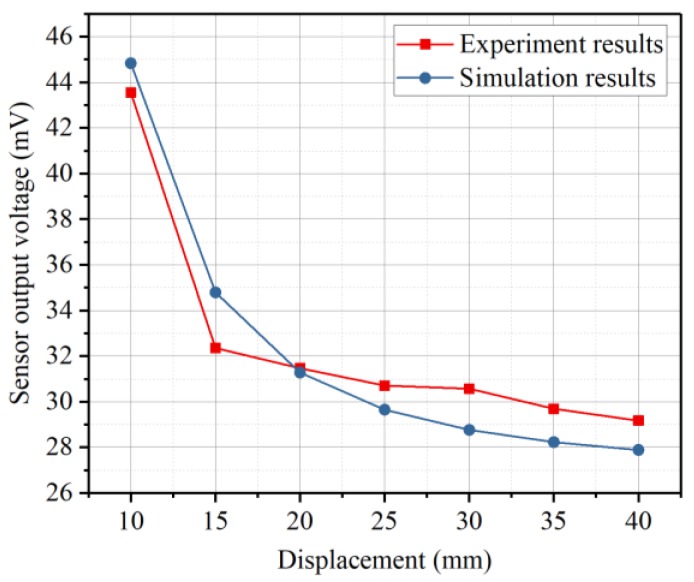
Results of signal obtained from the sensor versus displacement between the sensor and the vibrating sphere.

The simulation results can be obtained by calculating the pressure generated by the vibrating sphere using the empirical model and transferring the pressure into sensor output voltage according to the finite element analysis. Figure 14 shows the results. The sensor output voltage gradually falls with respect to the increasing of distance from the center of the sphere to the surface of the diaphragm.

The underwater vibrating sphere can generate the dipole flow field. The dipole flow field can be described by a velocity potential *Φ* with *v* = ∇*Φ*. For a sphere at position (0, *D*) that vibrates parallel to the *x*-axis and in the plane of the sensor diaphragm, the velocity potential can be described as [32]:

(3) $$\Phi (x,y,t)=\frac{-\mu (t)}{4\pi }\left\{\frac{x}{{\left[x^{2}+{\left(y-D\right)}^{2}\right]}^{3/2}}+\frac{x-D}{{\left[x^{2}+{\left(y+D\right)}^{2}\right]}^{3/2}}\right\}$$

(4) $$\mu (t)=2\pi a^{3}U\sin(\omega t)$$

where *a* is the diameter of the vibrating sphere, *U* is the velocity of the vibrating sphere, *ω* is the frequency of the vibrating sphere. The resulting water velocity on the surface of the sensor diaphragm is:

(5) $$v_{x}(x,y=0,t)=\frac{\partial \Phi }{\partial x}=\frac{\mu (t)}{2\pi }\frac{2x^{2}-D^{2}}{{\left(x^{2}+D^{2}\right)}^{5/2}}$$

The water flow velocity is linear with the velocity of the vibrating sphere. According to the Bernoulli Principle, the flow velocity on the topside surface of the sensor diaphragm will lead to a pressure difference across the diaphragm which can make the diaphragm a deformation and generate output voltage. When the disturbance generated by the vibrating sphere increases, the output voltage of the sensor will increase as well. Besides, the distance from the vibrating sphere to the sensor has a profound influence of the flow velocity on the surface of the diaphragm, hence the sensor output will decrease with respect to the increasing of the distance.

## 5. Conclusions

A sensor based on the radial field bulk piezoelectric diaphragm with inter-circulating electrodes is designed for detecting underwater disturbances. Finite element analysis including static analysis and modal analysis was conducted to simulate the polarization distribution of the diaphragm and estimate the behavior of the diaphragm. The electrodes on the diaphragm were fabricated using microfabrication technology. The impedance spectrum of the fabricated sensor which is examined by an impedance analyzer shows that the fundamental resonance frequency of the diaphragm is 14.45 kHz, and the diaphragm has an effective coupling coefficient of 5.51% and a quality factor of 32.84 (Dimensionless). Characterization of the sensors for oscillatory flow velocity sensing was performed underwater by employing a vibrating sphere as the stimulus. The results show that the sensor has a high sensitivity of 1.16 mV/(mm/s) at 35 Hz water flows, and a good response to the low frequency disturbance. This sensor has many advantages such as miniature size, light weight, self-powered, and low cost etc. It provides a good prospect for AUVs to improve the energy efficient sensing and maneuvering abilities.

## Figures and Tables

**Table 1:** Performance comparison of different PZT sensors.

Sensor	Size (Thickness × Diameter or Length)	Sensitivity	Fundamental Frequency	Coupling Coefficient	Quality Factor
Our design	300 μm × 10 mm	0.089 mV/Pa	14.45 kHz	5.51%	32.84
By Wang, et al. in [28]	27 μm × 6.6 mm	6.5 μV/Pa	15.69 kHz	4.91%	37.7
By Wang, et al. in [29]	7 μm × 0.5 mm	——	260.8 kHz	1.42%	——
By Polcawich, et al. in [30]	1 μm × 0.75 mm	0.92 μV/Pa	——	——	——
By Muralt, et al. in [31]	2 μm × 0.27 mm	——	752 kHz	5.3%	——
